# Towards reliable whole genome sequencing for outbreak preparedness and response

**DOI:** 10.1186/s12864-022-08749-5

**Published:** 2022-08-09

**Authors:** David F. Nieuwenhuijse, Anne van der Linden, Robert H. G. Kohl, Reina S. Sikkema, Marion P. G. Koopmans, Bas B. Oude Munnink

**Affiliations:** 1 Viroscience Department, Erasmus Medical Center, Rotterdam, the Netherlands; 2 Departement of Virology of the Vaccination Programme, RIVM, Bilthoven, the Netherlands

**Keywords:** Whole genome sequencing, Metagenomics, Arbovirus, Platform comparison, Long reads, Short reads, Epidemiology

## Abstract

**Background:**

To understand the dynamics of infectious diseases, genomic epidemiology is increasingly advocated, with a need for rapid generation of genetic sequences during outbreaks for public health decision making. Here, we explore the use of metagenomic sequencing compared to specific amplicon- and capture-based sequencing, both on the Nanopore and the Illumina platform for generation of whole genomes of Usutu virus, Zika virus, West Nile virus, and Yellow Fever virus.

**Results:**

We show that amplicon-based Nanopore sequencing can be used to rapidly obtain whole genome sequences in samples with a viral load up to Ct 33 and capture-based Illumina is the most sensitive method for initial virus determination.

**Conclusions:**

The choice of sequencing approach and platform is important for laboratories wishing to start whole genome sequencing. Depending on the purpose of genome sequencing the best choice can differ. The insights presented in this work and the shown differences in data characteristics can guide labs to make a well informed choice.

**Supplementary Information:**

The online version contains supplementary material available at 10.1186/s12864-022-08749-5.

## Background

Due to the increased connectivity of the modern world, deforestation and climate change, viral pathogens which used to be restricted to certain geographic areas or hosts have increased potential to spread to previously naïve populations. This is especially true for arthropod-borne (arbo) viruses like members of the genus *Flavivirus* as could be seen during the large Zika virus (ZIKV) outbreak in Brazil [1] and the recent expansion of Usutu virus (USUV) and West Nile virus (WNV) to Western Europe [2–5]. In Europe, also the risk of the introduction of other *Flaviviruses* like Yellow Fever virus (YFV) increases due to the expanding establishment of competent vectors along with other factors [6].

Whole genome sequencing (WGS) is increasingly advocated as important public health tool and has proven to be valuable during viral outbreaks to identify transmission chains, determine epidemic links and detect specific mutations [1, 7, 8]. Especially in the beginning of outbreaks this information may help to inform public health officials, provided that the data is generated and analysed in a timely fashion as was done for the recent SARS-CoV-2 outbreak [9]. This, however, can be challenging sometimes as it was for the Zika virus outbreak [10]. The successful and timely generation of WGS depends on the types of infections, sample types, instruments used for sequencing, costs, and quality of data and analysis. For instance for Zika virus, the viral load decreases rapidly after onset of symptoms [11], a phenomenon commonly observed during infections by members of the *Flaviviridae* family [12, 13] requiring protocols that work with low viral loads. The same applies when trying to sequence viruses from small volume samples, for instance when specimens from birds or mosquito pools are used [4].

During the more recent virus outbreaks, amplicon-based approaches were used to generate full length sequences of emerging viruses [1, 7, 9, 14]. This approach is specific and highly sensitive up to a Ct value of around 35 [15]. Nonetheless, the main limitation of this approach is that specific primer sets have to be developed for different (sets of) pathogens which are based on our current knowledge about virus diversity. This is not the case when using metagenomic sequencing, where all RNA and/or DNA present in the sample will be sequenced. However, this approach is sensitive to the presence of host background and/or bacterial DNA, decreasing the detection limit [16]. Capture probes can be used to increase the sensitivity of the metagenomic approach while still benefiting of the broad coverage of virus diversity. These probe sets can be designed to target a large spectrum of genomes of viral taxa that are known to infect vertebrates, thus providing potential to detect a wide range of pathogens. Briese et al. showed that a capture probe set (VirCapSeq-VERT) resulted in a 100- to 10,000 fold increase in viral reads compared to direct metagenomic sequencing [17].

There are several high-throughput second and third generation sequence machines available at the moment. The widely accepted golden standard is the sequencing by synthesis platform developed by Illumina, but novel platforms have been developed such as nanopore based sequencing by Oxford Nanopore. There are several differences between Illumina and Nanopore based sequencing. Compared to the Illumina sequencing machines, the cost of the Nanopore sequencing hardware is relatively low, the machine is portable, and data is generated in real-time. This gives Nanopore sequencing a benefit over Illumina sequencing in a setting where costs need to be kept to a minimum and speed is key [18]. However, the sequence method of choice is also dependent on the specific research question. For example, for early detection in the beginning of an outbreak, time to result is an important parameter, while later in the outbreak more detailed analysis using high quality sequencing reads to identify minority variants within patients may become important. For this application deep coverage with high quality reads can be preferred over speed.

The reported high error rate [19] compared to Illumina might limit the application of Nanopore sequencing, depending on how the data is analysed. For WGS the error rate can be compensated by creating a consensus sequence based on a larger number of overlapping reads compared to what is standard for Illumina sequencing. Previously it was shown that a read coverage of 100x is sufficient to compensate for the errors generated by Nanopore sequencing when using an R9.4 flowcell [20], which can go down to 20x using the recently released R10 flowcell [21].

**Fig. 1:**
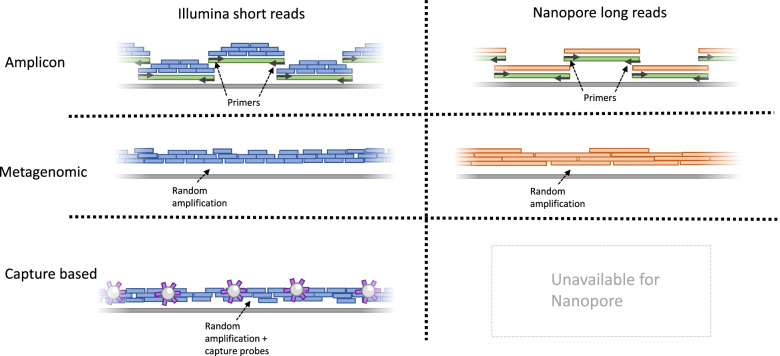
Overview sequencing approaches. Grey bars represent the to be sequenced genome, blue and orange are short and long reads respectively, generated either directly or from amplicons (green) or captured nucleic acid using capture probes (purple)

Here, the performance of whole genome sequencing is compared for four members of the *Flaviviridae* family in three different concentrations using five different sequencing approaches. Cell culture supernatants of USUV, WNV, YFV and ZIKV were diluted to a Ct value of 25, 29 and 33 and sequenced on the Illumina and Nanopore sequencing platform. The samples were sequenced using an amplicon-based approach and a metagenomic approach on both platforms and, due to technical constraints, a capture-based approach on Illumina only (Fig. 1).

## Results

### Amplicon based sequencing on the Nanopore and Illumina platform

**Fig. 2:**
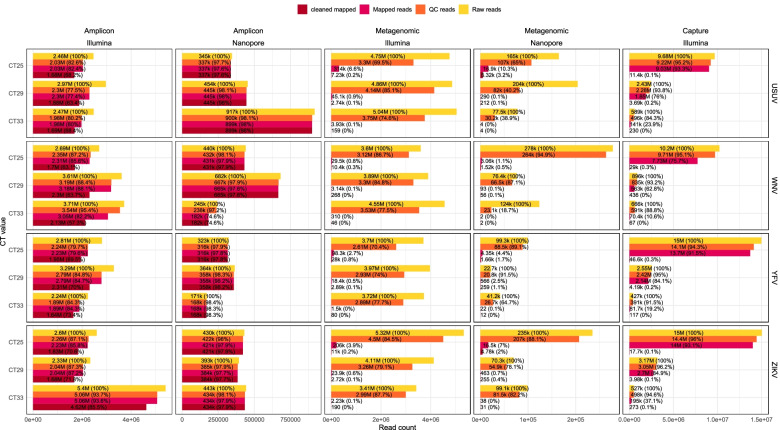
Overview of read numbers by virus and sequencing approach. The bars indicate the total number of sequence reads at different stages of the analysis process. Values and percentages of the number of reads are indicated in text, abbreviated using SI units

**Fig. 3:**
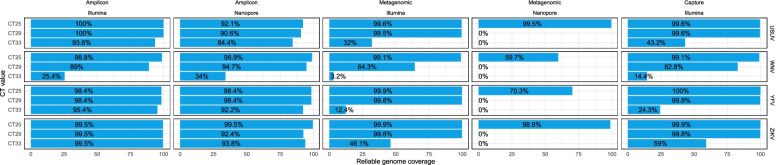
Overview of the percentage of reliable genome coverage. The bars indicate the percentage of coverage of the respective viral genomes with the respective approach and platform. The coverage percentage of amplicon data is calculated based on the amplified region of the genomes. The other percentages are calculated based on the size of the complete reference genome

**Fig. 4:**
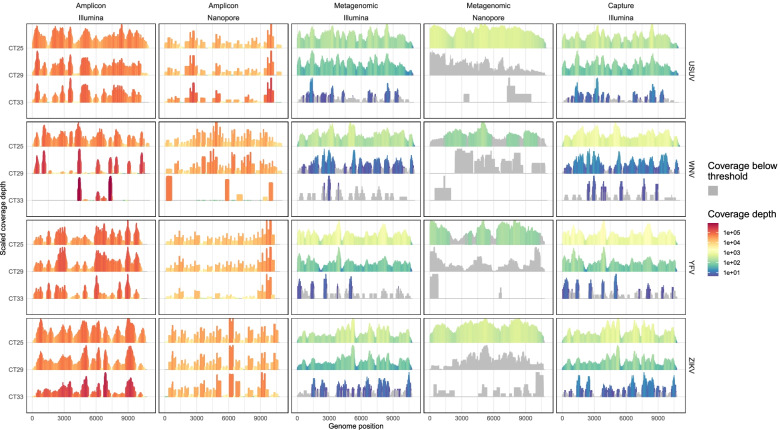
Overview of read coverage across the genomes. The height of the peaks represents the relative coverage profile at that position scaled by the maximum coverage of across the sample. The color scale represents the log transformed coverage depth. Profiles and coverage depth represent the cleaned mapping results. A grey coverage color indicates coverage below the coverage threshold (5x Illumina, 100x Nanopore)

When generating complete genomes using amplicon sequencing, there was little difference between Nanopore and Illumina. As expected, most reads belonged to the targeted virus (Fig. 2). On average, the total number of reads did not vary much for the different Ct values. Read counts for Illumina sequencing were around 3 M per sample with a single 5,4 M exception, while Nanopore produced around 400 k reads with a 682 k and 917 k as exceptions. Using the amplicon approach, both sequencing platforms were able to generate complete or near complete genomes from most samples. Illumina sequencing resulted in more or equal percentage coverage in most cases (10 out of 12), but performed worse in some cases (2 out of 12): for WNV Ct 29 and Ct 33 Nanopore covered 5 and 9% more of the genome respectively (Fig. 3). In all the amplicon based results the coverage depth varied greatly along the genome resulting in a spiky pattern which was mostly caused by differences in the performance of individual amplicons. In all cases the coverage difference between performant amplicons and failing amplicons was more than 51,000x (Fig. 4). Extreme differences in coverage were especially visible in high Ct samples such as Illumina WNV Ct 33 where one part of the genome had 800,000x coverage and other parts were not covered at all.

### Metagenomic sequencing on the Nanopore and Illumina platform

With the metagenomic approaches, using Nanopore sequencing resulted in, on average, 0.8% more virus specific reads than using Illumina, on an average of 1.8% viral reads (Fig. 2). The total number of reads per sample was heavily influenced by Ct value for Nanopore but not for Illumina, which produced around than 3 million reads for all samples. With Nanopore sequencing, the total number of reads dropped with 25% on average between the highest and the lowest Ct value, despite the equimolar pooling of the samples. Illumina sequencing resulted in reliable (more than 5x coverage) complete genome sequences for all samples with Ct 25 and 29 except for WNV. The samples with Ct 33 had between 3 and 46% of the genome covered at at least 5x. With Nanopore sequencing only the highest viral load USUV and ZIKV samples resulted in reliable (100x coverage) complete genome sequences. For the other viruses there was only partial coverage of the genome and the Ct 29 and Ct 33 samples had little to no coverage, but did allow idenficitation of all the viruses. The coverage profile of the Ct 25 Nanopore genomes was relatively smooth with high coverage across the entire genome. The coverage of the genomes generated by Illumina was less smooth resulting in an occasional drop of coverage below the 5x coverage threshold (Fig. 4).

### Capture-based sequencing on the Illumina platform

When using a capture-based approach for Illumina sequencing (here VirCapSeq-VERT) the percentage of viral reads was much higher compared to the metagenomic approach and comparable to the amplicon based approach. The total number of sequence reads was heavily influenced by the viral load in the sample as a result of the pooling all samples before capture (Fig. 2). The number of generated complete and near complete genome sequences was comparable to the amplicon-based Illumina approach although it performed worse for samples with Ct 33 (Fig. 3). The coverage profile of the genomes was relatively even, although some regions seemed to be preferably sequenced, resulting in coverage spikes. These spikes are especially noticeable in the Ct 33 samples and resemble the metagenomic Illumina coverage profile (Fig. 4).

### Influence of virus concentration and sequencing approach on consensus sequence quality

**Fig. 5:**
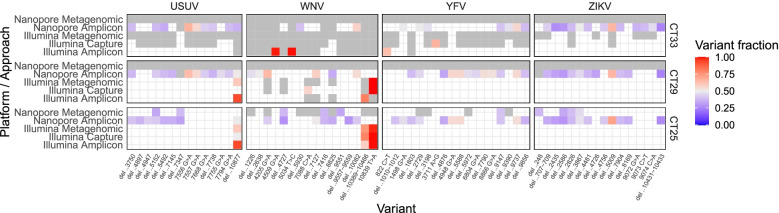
Overview of variants across the genomes. The x axis shows the variants found across the 4 genomes. The color scale represents the fraction of mapped reads containing the indicated variant in the read alignment. A darkgrey tile color indicates coverage below the coverage threshold (5x Illumina, 100x Nanopore)

The quality of the complete and partial genomes retrieved from the different sequencing approaches was evaluated by comparing the individual sequence variations found with each method. Using PySam [22], the major variants were extracted at each position in the alignment. Those variants that were present across all approaches (except for those that did not have sufficient coverage at the variant position) were accepted as true variants (Fig. S2). The other variants were considered errors and where investigated (Fig. S1). Most errors could be traced back to poorly trimmed primer sequences. Therefore we developed a custom script to better trim the primer sequences from the BAM file using the primer’s coordinates. In addition, several errors were found in the consensus sequences generated with the metagenomic or capture approach presumably resulting from PCR amplification errors. Dereplicating the reads in these alignments using Sambamba’s “markdup” method [23] resolved these errors, but reduced the number of mapped reads with 88% on average, showing that with these approaches many technical replicates are generated. Strongly softclipped reads (> 10%) were also removed as these were not dereplicated by Sambamba and often contained errors. After resolving these issues the remaining errors seemed to be related to the sequencing technology and the viral load (Fig. 5). Nanopore sequencing has difficulty with calling insertions and deletions, as multiple single nucleotide deletions were found, albeit at low variant fraction, resulting from the variable number of deletions present in the reads at these positions. For the same reason it was difficult to automatically call the large deletion at position 10,390 in the WNV genome. Also, several erroneous substitutions were found in the Nanopore data at a low frequency, which could be attributed to the error rate which caused systematic errors in some positions that were difficult to distinguish from real variants, especially since some of the true variants were also present at a relatively low frequency in the Nanopore data (Fig. S2). The only errors in the Illumina results were five false positive substitutions at positions WNV 4509, 5034, 7088 and YFV 822, 3711 (Fig. 5). These errors could not be attributed to primer errors, PCR amplification, or softclipping, but were located in low coverage areas of high Ct value samples indicating that the coverage may be too low for reliable variant calling with high Ct value samples.

The effect of these few errors on the interpretation of the genome sequences may seem unimportant, but can be crucial when using WGS for analyses that rely on the detection of only several variants, such as source tracing, lineage assignment and vaccine escape. In those cases, great care should be taken to rule out any false positive or false negative variants in the genomeby manual curation, especially at high Ct values. The errors in the Nanopore data are even more complicated to resolve automatically because real variants and erroneous variants can have similar variant frequencies. Especially insertions and deletions are hard to correctly interpret automatically and manual curation is necessary in most cases.

### Usability of different sequencing approaches for public health decision making

The use of the different sequencing approaches was also evaluated based on the time to result, the costs of the sequencing instrument, the costs per sample sequenced, the specificity, the suitability for sensitive WGS, and the application for initial detection of a pathogen (Table 1). Both the metagenomic and the capture-based approach can be used for initial detection of an unknown pathogen in the early days of an outbreak. Metagenomic Illumina sequencing is more suited for samples with lower viral loads, but the sequencing takes much longer and is more expensive. The cost and duration of metagenomic data analysis are also higher and longer, because assembling and annotating the obtained genomes is much more complex without a known reference genome. The preferred approach for contact tracing to identify transmission chains, or other situations that require quick results, is the amplicon-based Nanopore approach which results in full genomes up to Ct29-33 and can be performed within 1 day with relatively low costs per sample. However, for this approach the target of interest should be known a priori. Contact tracing with amplicon sequencing on the Illumina platform is more sensitive, but also more expensive and slower. Compared to metagenomic sequencing the analysis of amplicon data can be performed much quicker, and is therefore cheaper, because it can be automated in a workflow specifically tailored to finding a virus of interest. If amplicon sequencing is not an option the other approaches can give reliable results, but only at high viral load or at a higher cost and turnaround time.

**Table 1:** Overview of the usability of the different sequence methods. The sequence method, platform, time to results, costs and suitability for public health decision making is indicated

Sequence method	Amplicon	Amplicon	Metagenomic	Metagenomic	Capture-based
**Platform**	Illumina	Nanopore	Illumina	Nanopore	Illumina
**Time to result**	3 days	< 24 hours	3 days	< 24 hours	8 days
**Costs of the instrument**^**a**^	€200.000	€1.000	€200.000	€1.000	€200.000
**Cost of the analysis**^**b**^	€100	€100	€600	€600	€600
**Costs per sample**	€200	€50	€200	€50	€300
**Virus specific PCR required**	Yes	Yes	No	No	No
**Ct threshold for WGS**	Ct29-33	Ct25-29	Ct29-33	Ct25-29	Ct29-33
**Ct threshold for detection**	Ct33	Ct33	Ct33	Ct29-33	Ct33

^a^Estimated costs are laboratory and/or country dependent

^b^Estimated costs are estimated based on a €100 per hour and are laboratory and/or country dependent

## Discussion

Recent studies have shown the value of real-time WGS in public health decision making during a pandemic [24] and how WGS provides a tool to close the gaps in our knowledge about the global diversity of animal infecting viruses [25]. With the increasing demand for timely generation of sequence data, choises have to be made between short and long read sequencing and several different available protocols. We compared five different sequencing approaches for the detection and WGS of four different arboviruses in three dilutions and assess the potential use of the different sequence platforms and protocols for public health decision making in different stages of an outbreak.

We show that for the initial detection of an unknown viral pathogen the metagenomic approaches and the capture-based approach can be used in samples with a Ct value up to 33. Looking at sensitivity, capture-based Illumina sequencing is slightly superior to metagenomic Illumina sequencing, which is reflected in the total number of recovered viral reads across all dilutions and viruses. This is different from what was seen before, where capture was shown to result in a higher increase of genome coverage with an increase up to 20-fold for instance in a blood sample spiked with WNV, suggesting that capture is perhaps more suited for those samples with a very high amount of background host DNA or RNA or that for low Ct-value samples individual capture experiments have to be performed. Capture-based Illumina sequencing was also the most expensive and time consuming approach. Between metagenomic Nanopore and Illumina sequencing there is a tradeoff between turnaround time and sensitivity where Nanopore sequencing was shown to be 3 times faster, but is the least sensitive for initial virus detection as it generates only a few reads at Ct 33 in contrast to Illumina. However, given that for initial virus detection a few reads are sufficient and speed of detection is of importance [26], Nanopore metagenomic sequencing would be preferable.

For detailed outbreak investigation using phylogenetic analyses the focus is to generate an as complete as possible genome. From a sheer data volume point of view this seems to strongly favor Illumina sequencing which resulted in 50 and 61 million sequence reads for metagenomic and capture-based sequencing while Nanopore sequencing resulted in 5.2 million sequencing reads for the metagenomic approach. However, to achieve 5x coverage of the complete viral genome not that much data is necessary and for a limited number of samples the amount of reads generated with an Illumina run may be excessive. Previously, a coverage of 400x was determined to be sufficient to perform minor variant analysis [27] showing the excess of coverage with Illumina sequencing in our experiment, which resulted in up to 200 times more coverage. Nanopore has the benefit of generating much longer reads, which means that with the same number of reads more nucleotides can be covered. This can be seen by comparing the difference in mapped read counts with the difference in mapped nucleotides between Illumina and Nanopore. At Ct25, metagenomic Illumina sequencing on a Miseq produced 15 times more reads on average than metagenomic Nanopore, but the total number of sequenced nucleotides was only 5 times larger.

To make Illumina sequencing more cost effective more samples can be pooled in a single run, however the different sequencing approaches are also influenced by the pooling strategy. Even though samples were pooled equimolarly, there was a discrepancy in the number of sequence reads generated at different viral loads in the metagenomic Nanopore and capture-based Illumina sequencing approaches. The difference between the highest and the lowest viral load samples for the two approaches were up to 2- and 35-fold respectively. For metagenomic Nanopore the difference were the result of pooling, which is challenging due to the varying sequence lengths in the sequencing library and small pipetting errors, and therefore a 2-fold difference is generally seen as acceptable. With capture-based Illumina sequencing, the difference is mainly an effect of capturing viral DNA after pooling of the sequencing libraries. In lower load samples the fraction of viral DNA is lower compared to background or host DNA resulting in less captured DNA and therefore an underrepresentation of the sample in the final sequencing library since the pooling is performed based on the total amount of DNA present in the sample. This issue of balancing samples can be overcome by using individual capture reactions but this will also drastically increase the price of sequencing. It has been shown previously that multiple samples can be pooled and sequenced simultaneously [28], however, to do so, the nucleic acid concentration has to be measured accurately, which is especially difficult in the field, or in samples with very low viral loads.

When the target virus is known a priori and the focus is on in-depth characterization of the complete viral genome the amplicon approaches have a clear advantage and allow generating high quality genomes up to Ct 33. Nanopore is shown to do so at a modest cost and in a timely manner when 12 samples are multiplexed in one Nanopore sequence run, while Illumina sequencing gives similar results but at a higher turnaround time and cost. The benefit of longer reads is less pronounced with amplicon sequencing because the amplicons have a set size. Although larger amplicons would be possible with Nanopore sequencing, the reason behing using smaller amplicons is the increase in sensitivity [15]. Updating the amplicon primer pool and finetuning the primer concentrations will potentially increase the sensitivity of this approach even further and lead to more even coverage.

## Conclusions

In this work we compare three sequencing approaches on two sequencing platforms using four arboviruses in three different dilutions and show the performance with respect to sensitivity and WGS completeness. The amplicon-based approach performed best for WGS in almost all cases given the assumption that the target virus is known upfront. Capture-based Illumina sequencing performed best at agnostic virus detection, although at a higher cost and lower turnaround time compared to metagenomic sequecing. Choosing a sequencing platform and approach is important for labs adopting genome sequencing, but depends on the stage of an outbreak and the to be answered questions in public health decision making. The data presented in this work offers a deep insight in the characteristics of each approach and help making this choice.

## Material and methods

### Sample preparation

All the cell cultured passaged viruses were obtained from the Erasmus Medical Center. Viruses were cultured in Vero cells and cell culture supernatant was diluted in USUV, WNV, YFV and ZIKV negative serum to Ct values 25, 29 and 33 as determined by specific real-time PCRs [29–32]. RNA was extracted and aliquoted in different batches to prevent additional freeze-thaw steps. For USUV the AS201700077 strain was used (MN122189.1), for WNV the B956 strain (AY532665.1), for YFV the YFV_t146a212_Jan19_ur strain (MK760665.1) and for ZIKV the SL1602 strain (KY348640.1).

### Library preparation for amplicon sequencing

RNA was transcribed into cDNA using random hexamers (Thermo Fisher) and ProtoScript II (NEB) after which a multiplex PCR was performed in two different reactions as described previously [15]. For USUV and YFV the same primer set was used as previously described [20, 29], for ZIKV and WNV new primer sets were developed. The primer sequences and concentrations are displayed in supplementary Table 1. For Illumina sequencing the KAPA HyperPlus Kit (Roche) was used, while for Nanopore sequencing the native barcoding genomic DNA Kit was used (Nanopore).

### Library preparation for metagenomic sequencing

RNA was transcribed into cDNA using random hexamers (Thermo Fisher) and SuperScript IV (Thermo Fisher) and dsDNA was generated using Klenow (NEB). For Illumina sequencing the KAPA HyperPlus Kit (Roche) was used with the following modifications. The adapters were 1:10 diluted and an extra AMPure beads (Beckman Coulter) wash step was performed after adapter ligation. For Nanopore sequencing the “Low input genomic DNA” with PCR kit was used following the manufacturer’s instructions, (SQK-PBK004, Nanopore) apart from an additional wash step that was performed after adapter ligation.

### Library preparation for VirCapSeq-VERT sequencing

After metagenomic sequence library preparation, as described above, all samples were pooled to a final concentration of 1000 ng and a specific capture for all vertebrate viruses was performed using VirCapSeq capture probes which were previously described [17]. All 12 samples were multiplexed in one capture reaction. The capture was performed according to the manufacturer’s instruction (Roche) and the hybridization reaction was incubated for 72 h.

### Illumina and Nanopore sequencing

For Nanopore sequencing the DNA concentration was quantified using the Qubit (Thermo Fisher) while for Illumina sequencing the DNA concentration was quantified using the KAPA Library Quantification Kit (Roche). The size of the library was determined on a Agilent Bioanalyzer using the Agilent High Sensitivity DNA kit. For all 5 different sequencing approaches, samples were pooled equimolarly and run on a single flow cell. Illumina sequencing was performed on an Illumina MiSeq to generate 2x300nt paired end sequences and Nanopore sequencing was performed on a GridION using R9.4 FLO-MIN106 flowcells with a run time of 16 h.

### Bioinformatic analysis

Nanopore sequences were demultiplexed using Porechop (https://github.com/rrwick/Porechop) after which the reads were trimmed to a median PHRED score of 10 and a minimal length of 150 nt using fastp [33]. Illumina sequences were trimmed from the 3′ end with a windowed approach and a mean PHRED score threshold of 20 using fastp [33]. Minimap2 [34] and BWA-MEM [35] were used to map the Nanopore and Illumina sequence reads respectively to MN122189.1 (USUV), AY532665.1 (WNV), MK760665.1 (YFV) and KY348640.1 (ZIKV). After mapping primer sequences were clipped with python script using PySam [22] and reads with more than 10% softclipped nucleotides were removed from the alignment. The coverage statistics were determined using samtools’s depth method [36]. A custom R script was used to generate the figures and determine the percentage of genome coverage above the coverage thresholds. For Nanopore sequencing the coverage threshold for reliable read coverage was set to 100x, as previously described [20], while for Illumina sequencing, because of its much higher read quality, the threshold was set to 5x read coverage. The complete workflow was written as a Snakemake [37] workflow which is, together with the custom python and R scripts, available at https://github.com/dnieuw/platform-comparison-arbovirus.

## Supplementary Information

**Additional file 1: Figure S1.** Overview of variants across the genomes before cleanup of read mapping. The x axis shows the variants found across the 4 genomes. The color scale represents the fraction of mapped reads containing the indicated variant in the read alignment. A darkgrey tile color indicates coverage below the coverage threshold (5x Illumina, 100x Nanopore). **Figure S2.** Overview of true variants across the genomes. The x axis shows the variants found across the 4 genomes. The color scale represents the fraction of mapped reads containing the indicated variant in the read alignment. A darkgrey tile color indicates coverage below the coverage threshold (5x Illumina, 100x Nanopore).

**Additional file 2.**

## Data Availability

All read data are publically available under PRJEB47177, scripts to reproduce the results of this study are available at https://github.com/dnieuw/platform-comparison-arbovirus.

## Abbreviations

Ct: Cycle threshold
USUV: Usutu virus
WGS: Whole genome sequencing
WNV: West Nile virus
YFV: Yellow Fever virus
ZIKV: Zika virus
