# Supplementary material for: Towards reliable whole genome sequencing for outbreak preparedness and response
Source: BMC Genomics. 2022 Aug 9;23:569. doi: 10.1186/s12864-022-08749-5 (PMC9361258; doi:10.1186/s12864-022-08749-5)
Supplement: Supplementary file 1 — Additional file 1: Figure S1. Overview of variants across the genomes before cleanup of read mapping. The x axis shows the variants found across the 4 genomes. The color scale represents the fraction of mapped reads containing the indicated variant in the read alignment. A darkgrey tile color indicates coverage below the coverage threshold (5x Illumina, 100x Nanopore). Figure S2. Overview of true variants across the genomes. The x axis shows the variants found across the 4 genomes. The color scale represents the fraction of mapped reads containing the indicated variant in the read alignment. A darkgrey tile color indicates coverage below the coverage threshold (5x Illumina, 100x Nanopore). [file 12864_2022_8749_MOESM1_ESM.zip › FigureS2.pdf]

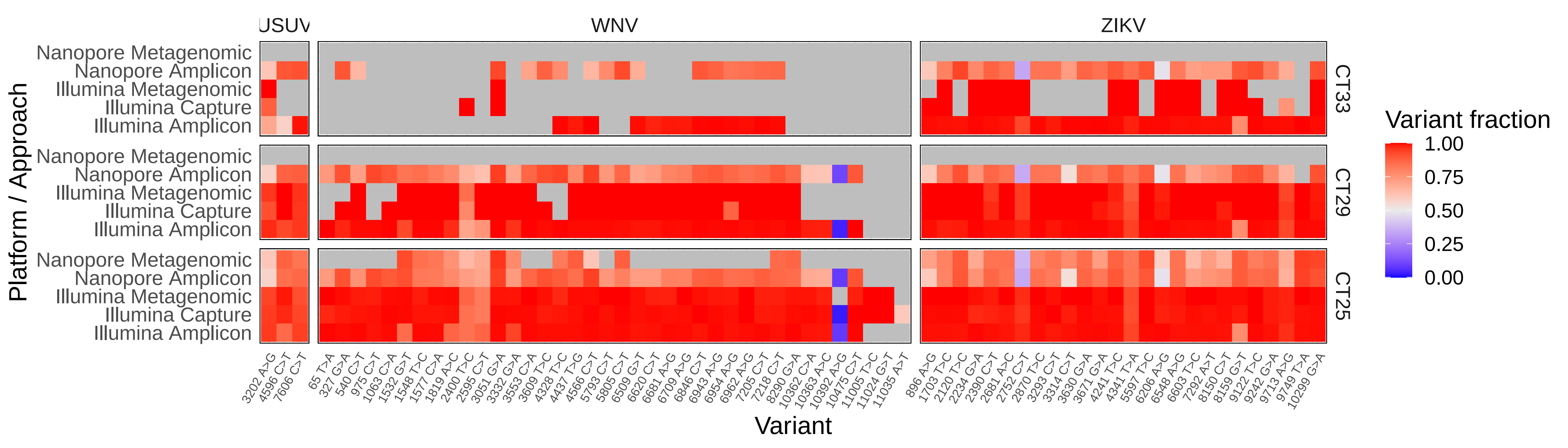

USUV
WNV
ZIKV
Platform / Approach
Nanopore Metagenomic
Nanopore Amplicon
Illumina Metagenomic
Illumina Capture
Illumina Amplicon
CT33
CT29
CT25
Variant fraction
1.00
0.75
0.50
0.25
0.00
3202 A>G
4596 C>T
7606 C>T
65 T>A
327 G>A
540 C>T
975 C>T
1063 C>A
1532 G>T
1548 T>C
1577 C>A
1819 A>C
2400 T>C
2595 C>T
3051 G>A
3332 G>A
3553 C>A
3609 T>C
4328 T>C
4437 T>G
4566 C>T
5793 C>T
5805 C>T
6509 G>T
6620 C>T
6681 A>G
6709 A>G
6846 C>T
6943 A>G
6954 A>G
6962 A>G
7205 C>T
7218 C>T
8290 G>A
10362 C>A
10363 A>C
10392 A>G
10475 C>T
11005 T>C
11024 G>T
11035 A>T
896 A>G
1703 T>C
2120 T>C
2234 G>A
2390 C>T
2681 A>C
2752 C>T
2870 T>C
3293 C>T
3314 C>T
3630 G>A
3671 G>A
4241 T>C
4341 T>A
5597 T>C
6206 A>G
6548 A>G
6603 T>C
7292 A>T
8150 C>T
8159 G>T
9122 T>C
9242 G>A
9713 A>G
9749 T>A
10299 G>A
Variant
