# Supplementary figures and images for: Towards reliable whole genome sequencing for outbreak preparedness and response
Source: BMC Genomics. 2022 Aug 9;23:569. doi: 10.1186/s12864-022-08749-5 (PMC9361258; doi:10.1186/s12864-022-08749-5)

Platform / Approach

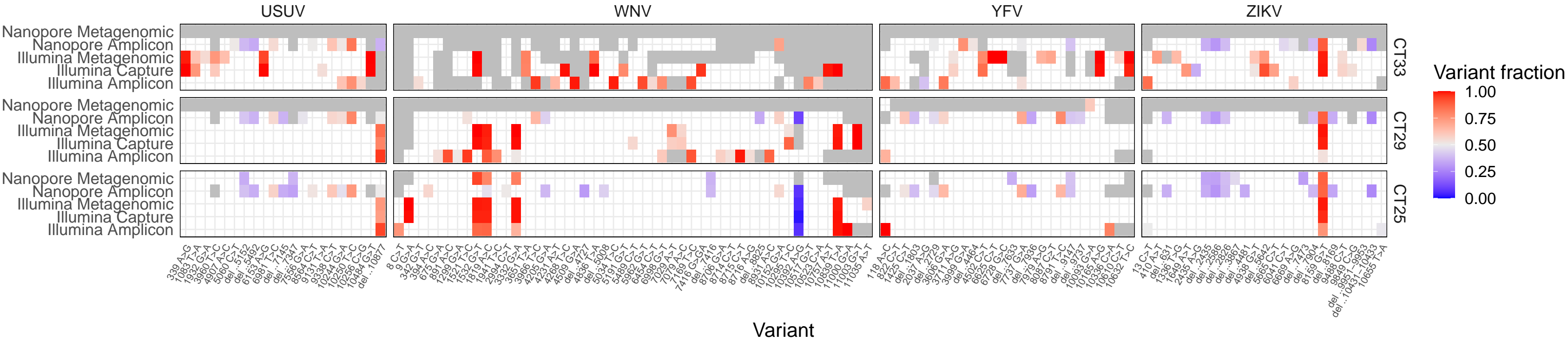

Supplement: Supplementary file 1 — Additional file 1: Figure S1. Overview of variants across the genomes before cleanup of read mapping. The x axis shows the variants found across the 4 genomes. The color scale represents the fraction of mapped reads containing the indicated variant in the read alignment. A darkgrey tile color indicates coverage below the coverage threshold (5x Illumina, 100x Nanopore). Figure S2. Overview of true variants across the genomes. The x axis shows the variants found across the 4 genomes. The color scale represents the fraction of mapped reads containing the indicated variant in the read alignment. A darkgrey tile color indicates coverage below the coverage threshold (5x Illumina, 100x Nanopore). [file 12864_2022_8749_MOESM1_ESM.zip › FigureS1.pdf]
